# Mapping Molecular Pathways of Multiple Sclerosis: A Gene Prioritization and Network Analysis of White Matter Pathology Transcriptomics

**DOI:** 10.1002/ana.27216

**Published:** 2025-02-14

**Authors:** Gianmarco Abbadessa, Ai Nagano, Simon Hametner, Owain Howell, David Owen, Artemis Papadaki, Prashant Srivastava, Simona Bonavita, Roberta Magliozzi, Richard Reynolds, Mie Rizig, Richard Nicholas

**Affiliations:** ^1^ Department of Advanced Medical and Surgical Sciences University of Campania Luigi Vanvitelli Naples Italy; ^2^ Department of Brain Sciences Imperial College London London UK; ^3^ Department of Neuromuscular Diseases UCL Queen Square Institute of Neurology London UK; ^4^ Division of Neuropathology and Neurochemistry, Department of Neurology Medical University of Vienna Vienna Austria; ^5^ Institute for Life Sciences Swansea University Swansea UK; ^6^ Centre for Inflammatory Disease, Department of Immunology and Inflammation, Faculty of Medicine Imperial College London London UK; ^7^ National Heart and Lung Institute, Faculty of Medicine Imperial College London London UK; ^8^ Department of Neurosciences, Biomedicine and Movement Sciences University of Verona Verona Italy

## Abstract

**Objectives:**

Rapid advances in transcriptomics have driven efforts to identify deregulated pathways in multiple sclerosis (MS) tissues, though many detected differentially expressed genes are likely false positives, with only a small fraction reflecting actual pathological events. Robust, integrative methods are essential for accurately understanding the molecular mechanisms underlying MS pathology.

**Methods:**

We conducted a gene prioritization analysis of MS white matter pathology transcriptomic studies. Articles were sought in Scopus and PubMed up to July 31, 2024. Potentially eligible publications were those that provided either transcriptomics datasets (deposited in GEO) or lists of differentially expressed genes comparing MS white matter to control white matter.

**Results:**

Applying a vote‐count strategy to search for the intersection of genes reported in multiple independent studies with a consistent fold‐change direction, followed by a Monte Carlo simulation, we identified 528 highly significant differentially expressed multi‐study genes (*p* < 0.0001; 10,000 simulations). Functional enrichment analysis revealed deregulation of the folate pathway in MS normal‐appearing white matter, and tumor necrosis factor (TNF) ‐related and complement‐related pathways in active and chronic active lesions, respectively. Network analysis identified 6 key signaling hubs: PTPRC, HLA‐B, MYC, MMP2, COL11A2, MAG. The major nodes identified revealed mechanistic concordance with published in vivo MS models, supporting their value as potential therapeutic targets.

**Interpretation:**

Our strategy provides a robust framework for integrating gene expression data, effectively identifying the intricate pathways altered in human diseased tissues. This method holds potential for translating findings into drug development strategies. ANN NEUROL 2025;98:67–79

Multiple sclerosis (MS) is a chronic inflammatory disease of the central nervous system (CNS), characterized by complex, diffuse, and varied tissue alterations.^1^ It is hypothesized that the peripheral immune system triggers inflammatory events that cause MS relapses and active demyelinating lesions, whereas more complex phenomena intrinsic to CNS tissue promote the compartmentalized inflammation contributing to neurodegenerative processes.^2^ The rapid advance in omics technologies over the past 2 decades has catalyzed efforts to identify deregulated pathways in MS tissues, aiming to delineate the molecular signature of MS pathogenesis.^3^ Most studies have focused on white matter (WM) lesions, providing lists of differentially expressed genes (DEGs), along with the enriched molecular pathways they implicate.^4^, ^5^, ^6^, ^7^, ^8^, ^9^, ^10^, ^11^, ^12^, ^13^, ^14^, ^15^, ^16^ Collectively, these studies have provided essential information that has guided subsequent experimental research in uncovering the mechanisms driving tissue damage. However, as is typical for these approaches, hundreds of the identified genes may be false positives, with only a small fraction reflecting pathological events, thus holding potential as biomarkers or therapeutic targets. These genes should be over‐represented across studies, while system‐specific non‐essential genes should be under‐represented.^17^, ^18^, ^19^ Reflecting this concept, the strategy of searching for the intersection of genes identified in multiple independent studies has become increasingly popular in other fields.^17^, ^18^, ^19^ However, due to challenges related to collecting data from brain tissue samples, the CNS disease field has lagged behind. Only in the past decade has there been a significant increase in the number of studies providing gene expression data from MS tissues, and they have not yet been integrated using a systematic and validated approach. Here, we integrated published evidence from multiple transcriptome datasets, applying a vote‐counting strategy based on the number of studies reporting a gene as consistently differentially expressed, followed by a Monte Carlo simulation, to (1) systematically identify highly significant multi‐study genes as signatures of MS WM pathology, (2) elucidate enriched molecular pathways and construct de novo networks from these significant genes, and (3) validate these candidates through published in vivo models to assess their suitability as therapeutic targets.

## Materials and Methods

**FIGURE 1:**
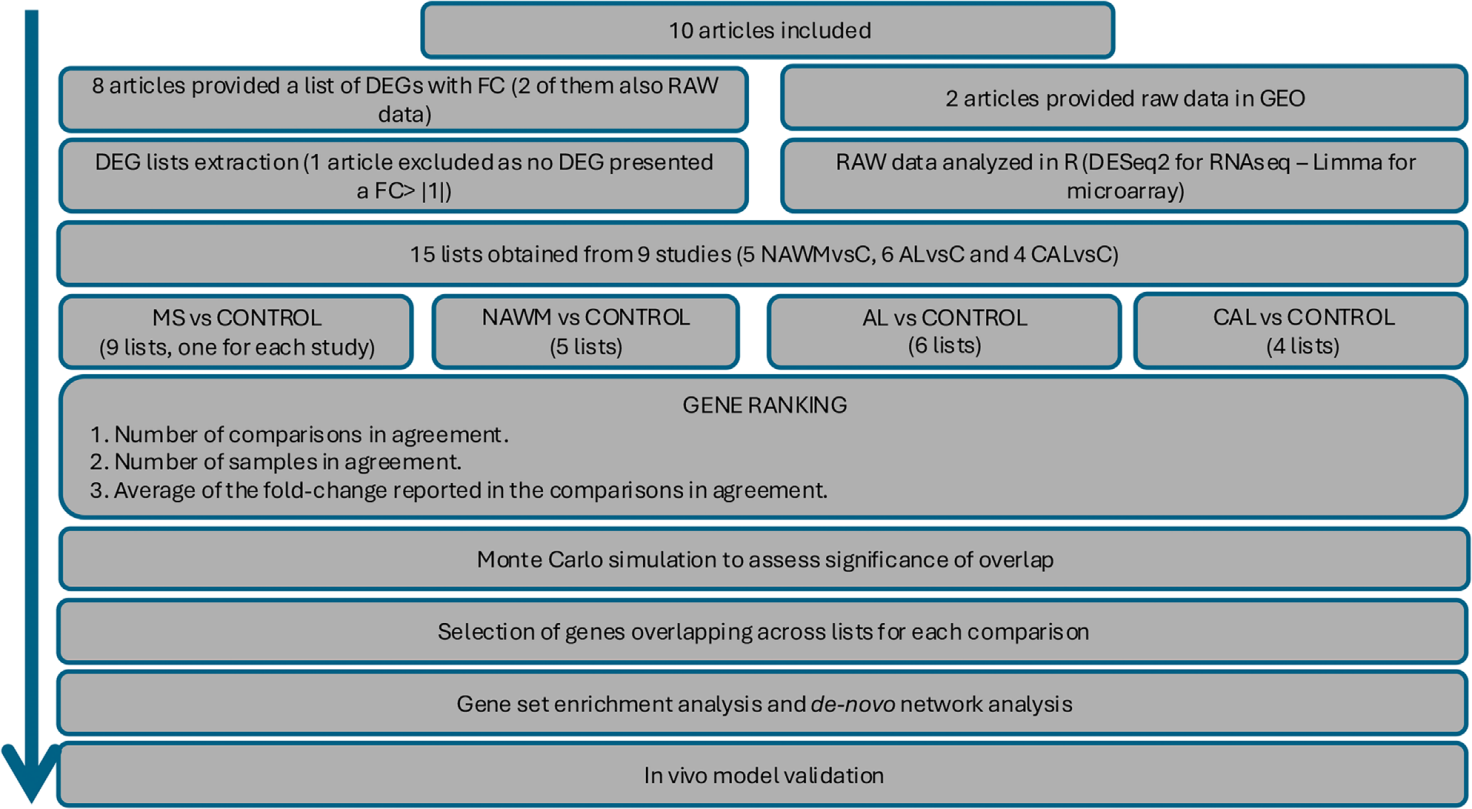
Study framework. Flow diagram illustrating the procedure employed to identify highly significant multi‐study genes and the resulting enriched pathways and de novo networks. [Color figure can be viewed at www.annalsofneurology.org]

The methodological procedure is represented in Figure 1.

### Protocol

The systematic review was performed following the guidelines of the Preferred Reporting Items for Systematic Reviews and Meta‐Analyses (PRISMA).^20^ To ensure the uniqueness of this review, databases such as PROSPERO, Scopus, Cochrane Library, and PubMed were thoroughly searched for existing similar work.

### Eligibility Criteria

In this review, the Population, Intervention, Comparators, Outcome, and Study design (PICOS) was adapted to focus on the following aspects: *Population* – MS brain donors; *Intervention* – neuropathological evaluation and gene expression analysis of brain tissue donors; *Comparators* – non‐MS brain donors; and *Study Design*: the review focuses on original, peer‐reviewed observational studies.

Articles were included if they met the PICOS stated criteria: Gene expression analysis was performed in MS WM and matched non‐MS WM tissues; Neuropathological assessment was performed to classify lesion stage; The MS sample group comprise at least 3 donors; and The study design was cross‐sectional.

Studies focusing on gray matter and choroid plexus, specific cell types, snRNAseq studies, spatial transcriptomic studies and those involving fewer than 3 MS brain donors were excluded. Non‐human, in‐vitro studies, case reports, editorials, commentaries, and reviews were excluded.

### Search Strategy

An electronic search for articles published from 1999 to March 2024 was conducted in PubMed and Scopus using categorized search terms. The search syntax was reported in supplementary file (Table S1). The complete search outcomes were examined through titles and abstracts on 2 distinct occasions by 1 reviewer. We expanded the search process by thoroughly reviewing the reference lists of the identified articles. Particular attention has been given to the reference lists of review articles. Eligible results were assessed against the eligibility criteria independently by 2 reviewers (Tables S2 and S3).

### Quality Assessment

To assess the quality of the included studies, we adapted the quality appraisal tool for cross‐sectional studies of biomarker data (BIOCROSS). We replaced some questions (in red in Table S4) to fit the experimental design and nature of the data related to brain tissue gene expression studies.

### Data Extraction and Analysis

Data of interest included first author, year of publication, sample size (in term of number of brain donors and number of tissues analyzed for each donor), brain donor demographics, tissue types and comparisons, methods used for assessing gene expression (reverse transcriptase‐polymerase chain reaction [RT‐PCR], microarray, or RNA‐seq) and platform, DEG list for each comparison performed in the selected studies, and GSE ID availability. DEG lists were extracted from the main text or Supplementary Materials of each study. The DEG lists extracted from the selected studies were, then, processed to filter genes with a log2 fold change >|1| and a *p* < 0.05. Exceptions: Baranzini et al. 2001 used a significant threshold of 2.5 (fold change), Lindberg et al. applied as threshold a *p* value <0.025; Melief et al. applied as threshold an adjusted *p*‐value using the Benjamini‐Hochberg method <0.05.

For 2 studies,^11^, ^16^ the lists of DEGs for each comparison were not available in full but the raw data were deposited in the GEO database, which we re‐analyzed. We performed the following comparisons: normal appearing WM (NAWM) versus control WM, active lesions (AL) versus control WM, and chronic active lesions (CAL) versus control WM for Elkjaer et al.^16^ and the following comparisons: NAWM versus control WM and CAL versus control WM for Hendrickx et al.^11^ The analyses were conducted in RStudio using the limma for microarray^11^ data and DESeq2 packages for RNA‐seq data,^16^ with the R code provided on the GEO platform (threshold for identifying DEGs fold change>|1|, adjusted *p* < 0.05).

For each DEG list, we obtained the following information: gene symbol (or other unique identifier when gene symbol was not available), fold change, *p* value, and false discovery rate (FDR) adjusted *p* value at 5%. The lists were grouped based on the comparison performed (NAWM vs control WM, AL vs control WM, CAL vs control WM).

### Gene Mapping

As a common target identifier, we chose the National Centre for Biotechnology Information's Entrez gene identifier. Gene symbol mapping to the respective Entrez ID was performed in RStudio, with the org.Hs.eg.db package. We manually mapped genes not recognized by the packages using Gene Card and NCBI Gene.

### Gene Ranking

Each study included provided lists of DEGs for 1 or more comparisons (e.g., NAWM vs control WM). We first evaluated the comparison between MS WM versus control WM (regardless of the lesion stage), then, we evaluated separately the comparison based on the different lesion stage (NAWM vs control WM, AL vs control WM, and CAL vs control WM). For the comparison between MS and control WM, we merged the lists of different lesion stage comparisons provided by the same study. After removing duplicates and keeping only the unique DE gene symbols in each study, we retained the one with the highest absolute fold change.

We did not conduct direct comparisons between MS tissue types (e.g., NAWM vs AL, NAWM vs CAL, or AL vs CAL). This decision was based on concerns about variability in tissue sampling and classification methods across studies, which could compromise both the reliability and rigor of such comparisons.

A vote‐counting strategy was employed to rank genes based on their relevance across study lists. This strategy was already employed in metanalysis of gene expression studies in other fields,^17^, ^21^, ^22^ showing that genes consistently reported in multiple datasets are more likely to reflect actual biological processes^17^ and, therefore, demonstrating a great validity in identifying high‐quality list of candidates.

This approach prioritizes genes consistently implicated across different datasets. The process of ranking the genes involved the following criteria, in order of relevance: (1) *Number of comparisons in agreement*: The number of lists (studies) in which the gene appears with the same fold‐change direction; (2) *Number of samples in agreement*: The sum of the samples from the comparison in agreement; and (3) *Average of the fold change reported in the comparisons in agreement*.

### Weighted Gene Co‐expression Network Analysis and Monte Carlo Simulations

To assess the statistical significance of the observed overlap among DEG lists extracted from the selected studies, we employed a weighted sampling Monte Carlo simulation approach. Previous studies have validated the significance of gene overlap across studies by randomly sampling genes from a comprehensive gene list representing the data tested in the included studies.^17^, ^19^ However, a purely random sampling of genes, without accounting for the inherent dependencies among them, may fail to accurately capture the biological context, potentially leading to an inaccurate estimation of the *p*‐value for the vote‐counting procedure. Therefore, to provide a robust and biologically meaningful *p*‐value, we incorporated gene co‐expression dependencies into the simulations. To assess gene dependencies, we employed Weighted Gene Co‐expression Network Analysis (WGCNA)^23^, ^24^, ^25^ using GSE138614 as a surrogate for the gene universe. The rationale and detailed explanation of this approach are provided in the supplementary file, Supplementary Methods section.

For the Monte Carlo simulation, gene lists of varying sizes—corresponding to the DEG lists from each included study—were sampled 10,000 times from the comprehensive gene universe. Weighted sampling was performed using the connectivity scores generated from WGCNA analysis. For each iteration, the number of overlapping genes across DEG lists was recorded to create an empirical null distribution of overlaps. The observed overlaps were then compared against these null distributions to estimate *p*‐values.

For the MS WM versus control WM comparison, we performed Monte Carlo simulations using both random and weighted sampling to assess the impact of gene co‐expression dependencies on the overlap across simulated lists.

### Functional Enrichment and De Novo Network Analyses

Predefined pathways were identified by importing the lists of DEGs that showed overlap across at least 2 studies to String Web Interface. Selected databases included GO terms, which provided insights into the biological processes, molecular functions, and cellular components associated with the DEGs. Additionally, pathway analysis was conducted using Reactome (protein interactions) and WikiPathways (gene‐centric pathways) databases to identify biological pathways and networks relevant to the genes under study.

De novo networks were constructed using the String Web Interface and MCODE in Cytoscape. First, the list of DEGs that showed overlap across at least 2 studies was inputted into STRING to generate a protein–protein interaction network, which was then exported from STRING and imported into Cytoscape. The MCODE plugin was applied to identify subnetworks using the following parameters: degree cutoff = 2, node density cutoff = 0.3, node score cutoff = 0.2, k‐core = 4, and max depth = 100.^26^ Subsequent analysis of these subnetworks was conducted in Cytoscape. Key hubs were identified based on the highest betweenness centrality values. GO enrichment analysis was performed using the STRING Web interface to further characterize the biological relevance of the identified hubs and related networks.

### Published In Vivo Model Validation

To systematically assess the mechanistic impact of candidate genes on experimental autoimmune encephalomyelitis (EAE) and chronic EAE (CREAE), and their potential as therapeutic targets, we conducted a systematic literature search using Europe PMC (https://europepmc.org/), encompassing all PubMed entries.^27^ This search aimed to identify studies where specific perturbations (e.g., knockout, overexpression, inhibiting treatment, or activating treatment) of our candidate genes were conducted in vivo, with clinically relevant outcomes reported (amelioration, exacerbation, or no effect on the model). Candidate genes were defined as nodes with a betweenness centrality greater than 0.5. We utilized a Python script to query the Europe PMC database, generating search queries that combined Human Gene Nomenclature Committee symbols for candidate genes, with keywords related to EAE and in vivo models. The search was restricted to titles and abstracts of primary research articles. For each result, we evaluated whether a specific perturbation of the queried gene was performed in vivo, including gene knockout or knockdown, overexpression, inhibiting treatment (such as blocking antibodies or receptor antagonists), and activating treatment (such as receptor agonists). This process identified multiple specific in vivo perturbations of candidate genes in CNS disease models, which were compiled into a data frame. The effect on MS animal model for each perturbation was classified as follows: genes were annotated as “detrimental to MS” if a reduction in gene activity or its product (knockout, knockdown, inhibiting treatment) led to amelioration of the model outcome, or increased activity (overexpression, activating treatment) led to exacerbation. Conversely, genes were classified as “beneficial for MS” if a reduction in activity led to exacerbation, or increased activity led to amelioration. Genes with perturbations that did not affect model outcomes were classified as “no effect.” This classification resulted in a tally of published experiments for each candidate gene, indicating their overall effect on MS, which was visualized using the ggplot2 package in R.

## Results

### Literature Search and Data Extraction Results

**FIGURE 2:**
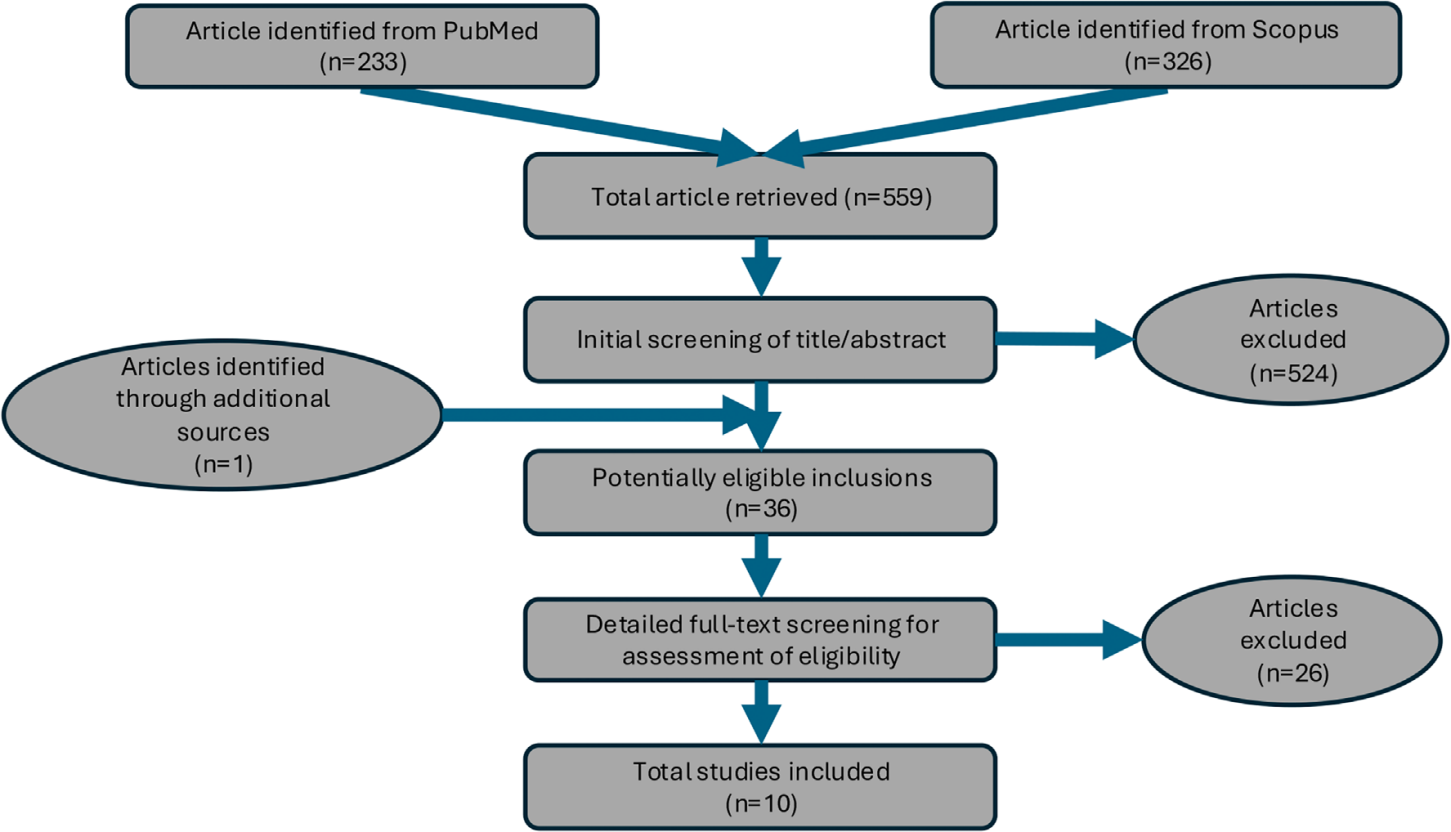
Preferred reporting items for systematic reviews and meta‐analyses (PRISMA) flow diagram. [Color figure can be viewed at www.annalsofneurology.org]

To integrate data from multiple transcriptome datasets, we, first, conducted a systematic review of gene expression profiling studies focused on MS WM pathology. In July 2024, the search returned 238 results from PubMed and 343 from Scopus, totaling 581 results. A preliminary review based on titles and abstracts led to the exclusion of 546 studies. Subsequently, 36 studies appeared potentially suitable for further examination, this included 1 study found in the references of Elkjaer et al (3). Ultimately, 10 studies met the inclusion criteria. Reasons for exclusion and details of excluded articles are detailed in supplementary file (Tables S3 and S4). The selection process is presented in a PRISMA flow diagram (Fig 2). Most studies provided more than 1 comparison; in detail, 6 studies compared NAWM to control WM, 6 studies compared AL to control WM, and 4 compared CAL to control WM (Table S5). Six studies quantified gene expression with microarrays, 1 study used express sequencing tag (EST), 1 study employed kinetic RT‐PCR,^4^ and the remaining RNA sequencing.^15^, ^16^

One study was excluded after the data extraction procedure^15^ as none of the genes reported in the list presented a fold change above 1 or below −1. A total of 15 comparisons were available from 9 studies. These studies consisted of 73 MS and 55 control brain donors, contributing a total of 193 WM tissue samples (Table S5).

Quality assessment results of the included studies are detailed in supplementary file (Table S4).

### Ranking Results

A total of 5,202 genes (3,369 upregulated and 1,773 downregulated; 60 with an inconsistent fold change) reported as being DEGs could be mapped to an Entrez gene identifier. In the comparison between control WM and MS WM, regardless of the lesion stage, 498 genes were reported in at least 2 studies with consistent fold changes. Twenty‐eight genes showed agreement across 3 studies, while only 2 genes, CASP1 and VIM, had a consistent fold change in 4 out of 9 studies. The NAWM versus (vs) control comparison resulted in 338 upregulated and 597 downregulated genes. The comparison between AL and control provided 2,241 upregulated genes and 974 downregulated genes, whereas for CAL, the number of DEGs identified was 3,227 (2,095 upregulated and 1,112 downregulated; 20 with an inconsistent fold change). The vote‐counting strategy resulted in 27 out of 938 genes with consistent fold changes in the NAWM versus control comparison, 49 out of 3,234 in the AL versus control comparison, and 250 out of 3,227 were reported in agreement in at least 2 studies in the CAL versus control comparison. The ranked gene lists are in supplementary file (Tables S6–S9).

### Monte Carlo Simulation

**FIGURE 3:**
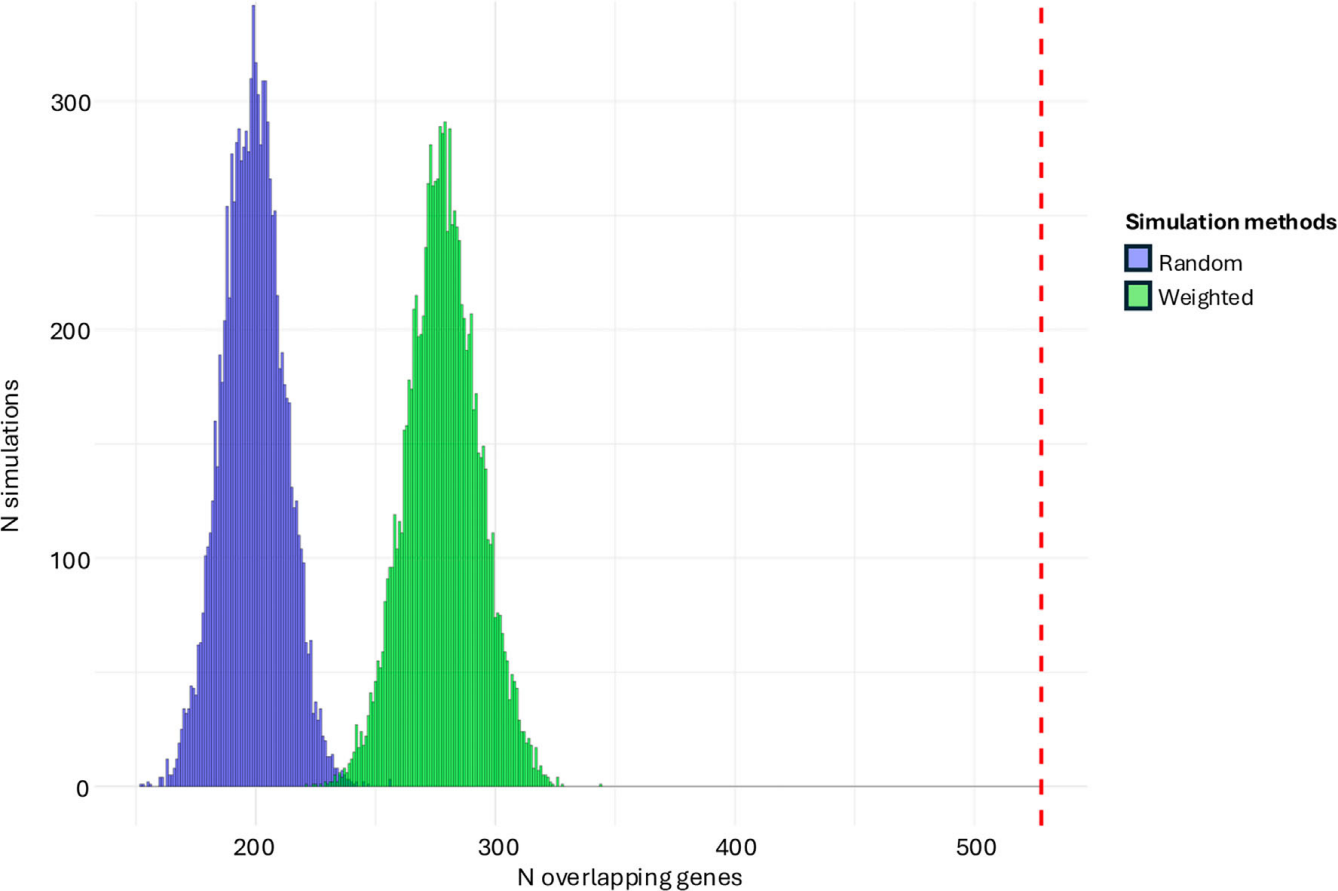
Distribution of simulated overlaps for multiple sclerosis (MS) versus control white matter (WM). Histogram displaying the frequency of overlaps observed in 10,000 simulations, with random sampling (*green*) and weighted sampling (*blue*) based on TOM‐based gene connectivity derived from Weighted Gene Co‐expression Network Analysis (WGCNA). Both simulated overlaps are significantly lower than the observed one (*p* < 0.0001). The red dashed line indicates the observed overlap (*n* = 528) across the studies. [Color figure can be viewed at www.annalsofneurology.org]

In all overlap analysis groups considered, except for AL versus control WM, we noticed a level of overlap found to be significant by Monte Carlo simulation (*p* < 0.0001; 10,000 permutations; Table 1). In the MS versus control comparison, 528 genes were found to be reported with the consistent fold‐change in more than 1 study (*p* < 0.0001; 10,000 permutations; Fig 3). In the simulation, an average of 267.39 (95% confidence interval [CI] 238.00–296.00) genes were reported with an overlap in 2, compared to 498 in our data (*p* < 0.0001; 10,000 permutations). The number of genes observed with an overlap in 3 studies was 28, compared to an average of 5.25 (95% CI 1.00–10.00) of simulated data (*p* < 0.0001). Two genes were consistently observed in 4 studies, compared to an average of 0.06 (95% CI 0.00–1.00) in the simulation (*p* = 0.0016).

**TABLE 1:** Monte Carlo Simulation Results

Comparison	N Comparison	Total Number of DEGs	N of DEGs with Multi‐study Confirmation	Simulated Overlap–Weighted Sampling	*p*‐Value
MS vs control	9	5,202	528	278.06 (SD 15.10)	<0.0001
NAWM vs control	5	938	27	10.09 (SD 3.08)	<0.0001
AL vs control	7	3,234	49	41.90 (SD 6.03)	0.13
CAL vs control	4	3,227	250	101.99 (SD 9.49)	<0.0001
MS vs control (sensitivity analysis)	5	5,064	492	237.07 (SD 13.80)	<0.0001

AL = active lesion; CAL = chronic active lesion; DEGs = differentially expressed genes; MS = multiple sclerosis; NAWM = normal appearing white matter; SD = standard deviation.

The overlap of genes resulting from the NAWM and CAL comparisons was highly significant (*p* < 0.0001; 10,000 permutations). No statistically significant difference was observed between AL versus control gene overlap and the simulated overlap. Two genes, DTNA and C1S, were observed to overlap in 3 studies in the CAL versus control comparison; however, the observation was not significant (*p* = 0.13) (Table 1). Further sensitivity analysis is reported in the Supplementary Material.

Two studies identified 4,131 and 1,201 DEGs (Elkjaer et al.^16^ and Hendrickx et al.,^11^ respectively), whereas other studies, limited by smaller sample sizes and older techniques, identified fewer than 150 genes each (Table S10). This imbalance skews the observed overlaps, with nearly 94% and 86% of the 528 overlapping genes derived from these 2 studies. In contrast, the other 7 studies contributed minimally, reflecting differences in technical depth rather than biology (Table S10). To mitigate bias, genes consistently identified in at least 2 studies were prioritized for functional enrichment and de novo network analyses.

### Functional Enrichment Analysis

#### Enriched Pathways from Upregulated Genes

Upregulated genes in MS WM, regardless of the lesion stage, led mostly to the enrichment of immune system related terms. The top 3 Gene Ontology (GO) biological processes terms were “Negative regulation of complement activation, lectin pathway,” “Positive regulation of antigen processing and presentation of peptide antigen via MHC class II,” and “Regulation of transcription from RNA polymerase II promoter in response to iron.” Among the numerous enriched pathways, “Classical antibody‐mediated complement activation” (Reactome), “Phosphorylation of CD3 and TCR zeta chains” (Reactome), and “Macrophage markers” (Wikipathway) were those with the highest strength. In NAWM, the only enriched pathway was “Folate Metabolism WP176” (Wikipathway). For AL, the identified pathways related to interleukin signaling (Reactome); several terms related to TNF signaling showed significant enrichment in the GO terms for Cellular Component and Molecular Function. In CAL, 9 out of the 19 identified pathways related to the complement cascade (Reactome and WikiPathway).

#### Enriched Pathways from Downregulated Genes

For the MS versus control comparison, regardless of the lesion stage, the analysis of downregulated genes resulted in the enrichment of “Central nervous system myelination” and “Oligodendrocyte development” as biological process and “Oligodendrocyte specification and differentiation, leading to myelin components for CNS” as a pathway (Wikipathway). A similar result was observed for CAL, whereas no enrichment was observed for NAWM and AL.

The lists of enriched terms and pathways are in supplementary file (Tables S11–16).

### De Novo Protein–Protein Interaction Network Analysis

The network analysis of the 528 identified MS WM DEGs led to the identification of 6 de novo protein–protein interaction networks and the following related hub genes: PTPRC, HLA‐B, MYC, MMP2, COL11A2, and MAG (Table 2). Overlapping genes across the lists of DEGs between NAWM and controls did not result in the enrichment of any de novo network. For AL, 1 network was identified (major hub‐gene: TGFB1). The analysis of CAL resulted in the identification of 2 de novo networks (major hub‐genes: CCL2 and GFAP) (Table 3). Connectivity and enrichment analysis results of the de novo identified networks are presented in full in Supplementary file (Tables [Link], [Link], [Link] and Fig S1–S9).

**TABLE 2:** De Novo Networks: Control WM versus MS WM

Network Figure	Nodes/Connections	Main Hub	Other Major Hubs	GO Function Enriched
Figure S1	199/1703	PTPRC (CD45)	ITGB2, CCL2, NCAM1, FCER1G	“Type III transforming growth factor beta receptor binding,” “Tumor necrosis factor binding,” and “MHC class II receptor activity.”
Figure S2	37/142	HLA‐B	HLA‐A, HLA‐DPA1, HLA‐DPB1, HLA‐DRB1	“MHC class II receptor activity,” “MHC class II protein complex binding,” and “Peptide antigen binding.”
Figure S3	132/400	MYC	PLEK, PPARG, HMOX1, CTSC	“Lipid binding,” “Signaling receptor binding,” “Identical protein binding”
Figure S4	91/217	MMP2	PLAU, CST3, SERPING1, LYZ	“Inhibitory MHC class I receptor activity,” “Metalloendopeptidase inhibitor activity,” “Serine‐type endopeptidase inhibitor activity”
Figure S5	27/53	COL11A2	COL8A2, COL8A1, COL9A2, SERPINH1	“Extracellular matrix structural constituent conferring tensile strength,” “Extracellular matrix structural constituent,” “Collagen binding.”
Figure S6	24/53	MAG	MOBP, OLIG2, MYRF, CNTN2	‐

Gene abbreviations are in Supplementary File S1.

AL = active lesions; CAL = chronic active lesions; DEGs = differentially expressed genes; GO = gene ontology; MS = multiple sclerosis; NAWM = normal appearing white matter; WM = white matter.

**TABLE 3:** De Novo Networks: Control WM versus AL and Control WM versus CAL

Network Figure	Nodes/Connections	Main Hub	Other Major Hubs	GO Function Enriched
Control WM vs MS AL				
Figure S7	21/72	TGFB1	MYC, IL15, NQO1, CCR5	“Chemokine (C‐C motif) ligand 5 binding”, “Superoxide dismutase activity”, “Tumor necrosis factor binding”
Control WM vs MS CAL				
Figure S8	60/213	CCL2	CD68, CASP1, CCR5, FCGR2B, FOLR2	“Chemokine (C‐C motif) ligand 5 binding,” “Cysteine‐type endopeptidase activity involved in apoptotic process,” “CARD domain binding”
Figure S9	25/55	GFAP	MAG, MOBP, OMG, GJC2	‐

AL = active lesions; CAL = chronic active lesions; DEGs = differentially expressed genes; GO = gene ontology; MS = multiple sclerosis; NAWM = normal appearing white matter; WM = white matter.

### In Vivo Models Validate a Role of the Main Hub‐Genes as Therapeutic Targets

To test whether our approach successfully identified causal genes suitable for therapeutic targeting, we conducted a systematic literature search for published in vivo experiments involving our candidate genes (nodes with a betweenness centrality higher than 0.5). Our search resulted in 124 identified studies that included combinations of our search terms in the title and/or abstract. Manual annotation of all publications was performed to select only those experiments where 1 of our candidate genes was specifically perturbed in vivo (i.e., knockout, overexpression, activating treatment, or inhibiting treatment) and a clinically relevant readout was reported (amelioration, exacerbation, or no effect on the model). We focused on EAE or CREAE, which represent the most common models for MS WM pathology.

**FIGURE 4:**
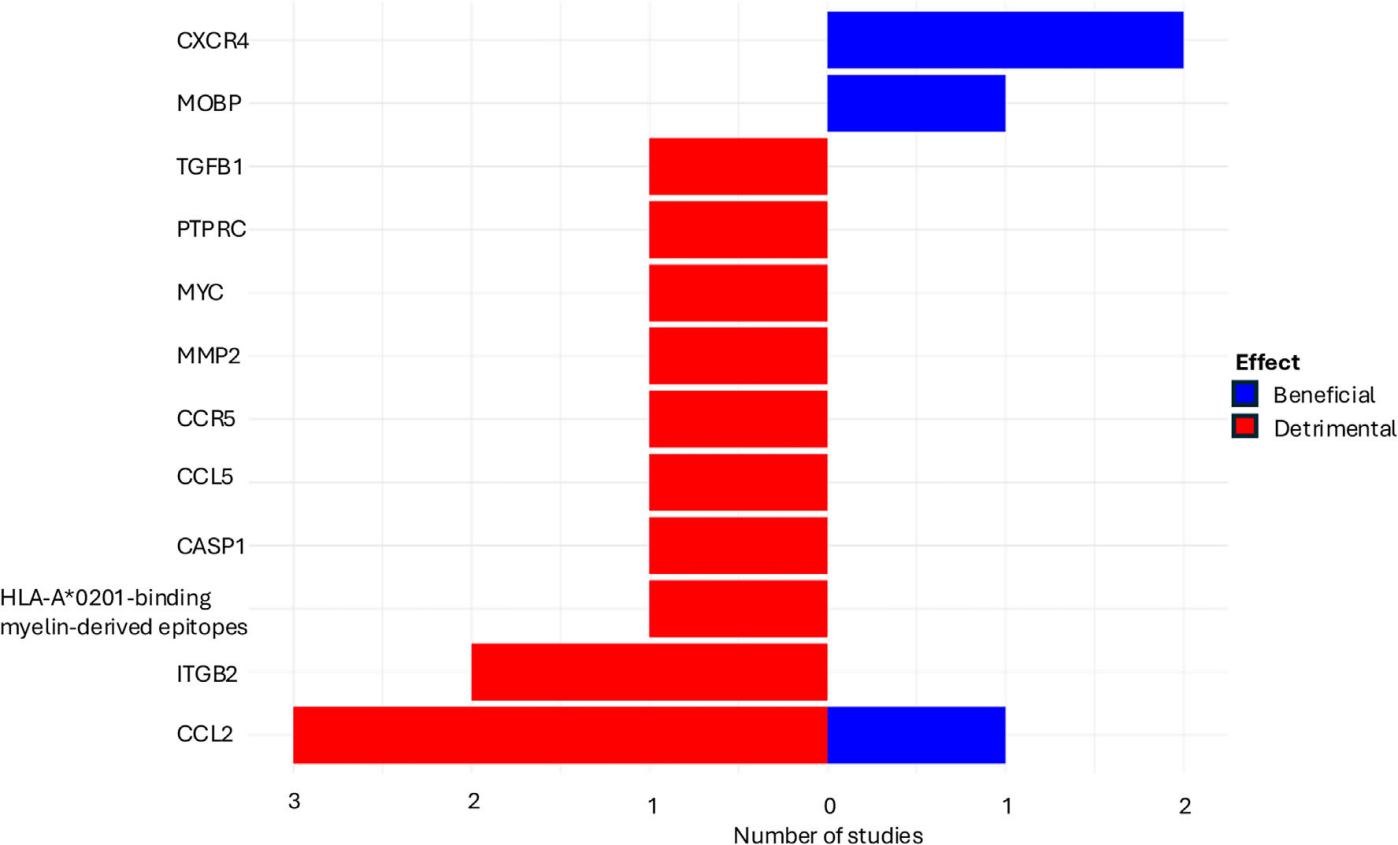
Effect of gene perturbations in experimental autoimmune encephalomyelitis (EAE) models. Bar chart illustrating the effects of candidate nodes perturbations on in vivo multiple sclerosis (MS) animal models. [Color figure can be viewed at www.annalsofneurology.org]

We identified 21 perturbational experiments for our candidate genes, involving 13 out of 38 (34.2%) candidate hubs (Fig 4 and Table S35). Only 2 out of 13 did not show a net positive or net negative effect on in vivo MS models (Fig 4 and Table S35). CCL2 emerged as a critical molecule with the highest number of perturbation experiments, demonstrating both beneficial and detrimental effects in EAE models. Low dosages of soluble CCL2 were found to delay EAE onset by downregulating Th1/Th17 cells and inducing regulatory T cells,^28^ while astrocyte‐CCL2 deletion led to a less severe EAE late in disease.^29^ Additionally, inhibition of CCL2 using *bindarit* ^30^ and mesenchymal stem cells‐derived CCL2 conversion to an antagonist^31^ were shown to effectively modulate disease progression. Therefore, targeting CCL2 with different cell specificities could potentially translate into a promising therapeutic target. Most of the candidates explored in in vivo perturbation experiments are proinflammatory and exclusively present evidence for detrimental effects on disease. The only exception was for the migration receptor CXCR4, which demonstrated a beneficial effect in 2 out of 2 experiments.^32^, ^33^ CXCR4 was shown to be necessary for mediating the beneficial effects of steroids and hyaluronic acid inhibitors in EAE models.^32^, ^33^

## Discussion

In the past 2 decades, researchers have made significant efforts to identify the molecular signature of MS pathology. Searching for the intersection of genes reported in multiple independent studies should identify those that consistently rise above the noise, indicating real biological effects.^17^, ^18^, ^19^ Adopting this strategy, we identified 528 highly significant genes in multiple studies and revealed critical pathway deregulations, such as the folate pathway in NAWM, and TNF‐ and complement‐related pathways in AL and CAL, respectively. Additionally, de novo network analysis pinpointed 6 key hub‐genes—PTPRC, HLA‐B, MYC, MMP2, COL11A2, MAG—highlighting their potential central roles in the disease molecular mechanisms.

**FIGURE 5:**
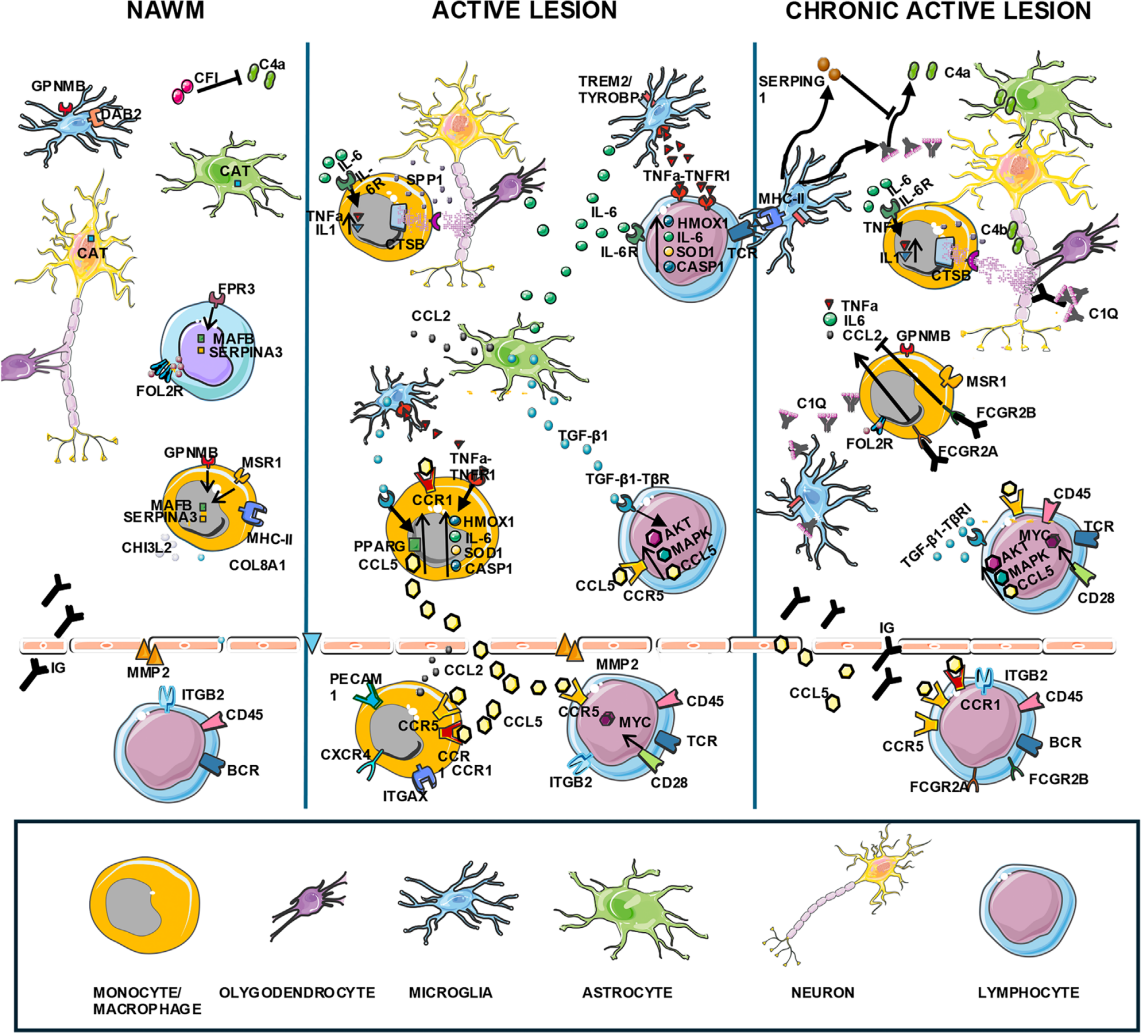
Key biological processes in multiple sclerosis (MS) white matter. (A) Normal appearing white matter (NAWM); The gene expression profile of NAWM highlights a complex dynamic involving the activation of immune processes and associated tissue damage, alongside the tissue efforts to protect, repair, and reduce inflammation. CD84, GPNMB, MSR1, and FPR3 indicate leukocyte activation, suggesting an ongoing inflammatory response. Upregulation of IGHG1 and IGKC points toward B‐cell activation and immunoglobulin production, while increased expression of CFI, DAB2, and MERTK indicates complement activity regulation and efferocytosis. Upregulated MAFB, COL8A1, and MMP2 suggest extracellular matrix remodeling, and changes in FOLR2, CAT, and SERPINA3 reflect altered folate metabolism and a protective response to oxidative stress. (B) Active lesion (AL). The initial event involves antigen presentation through MHC class II molecules and CD28 co‐stimulation, activating T cells and triggering immune responses. Pro‐inflammatory pathways involving TNF, IL‐6, CCL5, and CCR5, along with the activation of the NLRP3 inflammasome (CASP1/CASP4) by CTSB, amplify immune cell recruitment and inflammation, contributing to CNS tissue injury. The TGFB1 pathway indicates tissue remodeling, while antioxidant enzymes (SOD3, SOD1, HMOX1) are actively involved in protecting against oxidative stress. The upregulation of c‐MYC (possibly activated by CD28 downstream signaling) further indicates the ongoing broad spectrum of responses, including immune regulation, cell survival, and attempts at cellular repair and proliferation. (C) Chronic active lesion (CAL). The deregulation of complement components (C1q, C4a) and SERPING1 suggests complement system activation, mediating tissue damage at the edge of CAL. FCGR2A and FCGR2B facilitate phagocytic clearance of immune complexes, potentially exacerbating inflammation. CCL2, CCR5, and CCR1 enhance immune cell migration and activation in the CNS, while CASP1 and CASP4 are key for inflammasome activation, leading to cytokine production and apoptosis. CD68, MSR1, TYROBP, and GPNMB indicate macrophage and microglia involvement in tissue damage. TGFBI indicates tissue repair and fibrosis interplay, and FOLR2 upregulation may reflect the attempts to counteract inflammation. The figure was created using Servier Medical Art. [Color figure can be viewed at www.annalsofneurology.org]

Figure 5 summarizes the key biological processes identified in this study and discussed below.

We observed enrichment of the folate pathway when considering the highly significant multi‐study genes resulting from NAWM versus control comparison. A variant in the methylene‐tetrahydrofolate reductase (MTHFR) gene (encoding an enzyme of the folate pathway) was identified among the 200 non‐MHC MS risk associated genes (*p* = 2.31e‐15).^34^ This strongly supports the biological relevance of this finding, suggesting a potential involvement of folate metabolism in the pathology of MS, and warrants further investigation.

The gene signature of CAL resulted in the enrichment of complement‐related pathways and innate immunity functions. The central role of microglia and the involvement of complement pathways in the inflammatory process occurring at the edge of CAL was recently reported by a single nuclei study,^35^ identifying the complement component 1q as a critical mediator of “microglia inflamed in MS,” a glial phenotype that demonstrated neurodegenerative programming. Consistent with these findings, our analysis showed a significant upregulation of C1QB, reinforcing its involvement in CAL mechanisms. Additionally, we observed the deregulation of several other complement proteases and receptors in this lesion stage in multiple studies, including SERPING1, C1QB, CFI, C1S, C4B, C1R, and CFD. Notably, C1S, a serine protease that is part of the C1 complex in the classical complement pathway, was upregulated in 3 out of 4 studies examined.^6^, ^11^, ^16^ This strongly supports the involvement of complement component 1 in the mechanisms driving inflammation at the edge of CAL.

De novo protein–protein interaction network analysis identified 6 major networks and related hub‐genes:

(1) PTPRC or CD45 (Leucocyte Common Antigen) is highly expressed in leukocytes^36^ and is vital for T‐ and B‐cell activation, cell interactions, and regulation of protein‐tyrosine kinases.^37^, ^38^ A point mutation in the genes was shown to affect mRNA splicing, leading to altered CD45 isoform expression on immune cells.^36^ This mutation has been associated with MS in several case–control studies and family analyses, demonstrating its significant yet variable influence across different populations, likely due to genetic or environmental factors.^36^ Further, the variant chr1:198573373, located near the PTPRC gene, was linked to MS with a *p*‐value close to the genome‐wide association study (GWAS) threshold (*p* = 10e‐7).^34^

(2) Variation within the HLA region exerts the greatest individual effect on MS risk. The primary genetic risk factor for MS is the HLA‐DRB1*15:01 allele, alongside complex effects from different allelic lineages and protective signals in the class I region.^34^ Our findings, identifying key network hub‐genes such as HLA‐B, HLA‐A, HLA‐DRB1, HLA‐DPA1, and HLA‐DPB1 in MS WM, support and extend the substantial body of literature on the importance of the HLA gene cluster in MS pathogenesis.

(3) Cellular‐myelocymatosis (c‐MYC), a crucial transcription factor, regulates immune cell function and is central to MS transcriptional signatures. The CD28/PI3K/c‐myc axis enhances glycolytic metabolism in CD4+ T lymphocytes, promoting inflammatory cytokine expression.^39^ A single nucleotide polymorphism (rs4410871) in the c‐Myc gene associates it with MS risk (*p* = 7.70e‐09).^40^

(4) Matrix metalloproteinase 2 (MMP‐2), part of the zinc‐dependent endopeptidases degrading extracellular matrix proteins, contributes to neuroinflammation and blood–brain barrier (BBB) disruption in MS.^41^, ^42^ Here, we observed the consistent upregulation of MMP‐2 gene in NAWM in 2 studies, suggesting that MMP‐2 may play a role in the early phases of tissue damage in MS.

(5) Collagen genes, notably COL11A2, COL8A2, COL8A1, and COL9A2, are deregulated in MS WM, indicating significant extracellular matrix remodeling in response to inflammation. Variants in COL26A1 and near COL3A1 weakly associate with MS, emphasizing tissue remodeling pathways in its pathology (*p* = 7.98E‐04 and *p* = 3.73E‐03, respectively).^34^

(6) Lastly, the downregulation of myelin‐associated proteins like MAG, MOBP, CNP, OLIG2, and MYRF highlights disturbances in oligodendrocyte function and myelination, central to MS.

To validate potential novel drug targets for MS from our findings, we examined the effects of specific gene perturbations in in vivo models of MS, which supported their causal role in MS pathology. The findings demonstrate that most candidate genes exhibit clear detrimental or beneficial effects, reinforcing their potential as targets for therapeutic intervention. Notably, many of these targets included chemokine ligands and receptors, or molecules involved in cell migration, such as CCL2, CXCR4, CCL5, and CCR5, some of which are already in drug development pipelines for human use.^43^, ^44^, ^45^ Of note, Kauffmann and colleagues applied a similar approach for in vivo validation of receptor‐ligand candidates identified in a spatial transcriptome study of the MS brain cortex.^27^ Among the validated genes, CCL2 had the most supporting evidence,^27^ underscoring the robustness of our approach.

Although our approach aimed to identify major hub genes central to MS molecular mechanisms through the combination of a vote‐counting strategy and de novo network analysis, the high cross‐study consistency of CASP1 and VIM is also noteworthy. These genes emerged as consistent DEGs in 4 out of 9 studies, a significant overlap suggesting their involvement in ongoing biological processes. VIM, an intermediate filament protein in astrocytes and endothelial cells, contributes to glial scar formation, stabilizing astrocytic structure to protect surrounding tissue while potentially limiting neuronal regeneration.^46^, ^47^ Its elevated expression may indicate a role of astrocytes in MS pathology, balancing damage limitation and repair within demyelinated regions. CASP1, crucial in inflammasome activation and pyroptosis via interleukin (IL)‐1β and IL‐18 release, impacts both microglia and oligodendrocytes. Studies in the EAE model show that targeting the NLRP3‐CASP1‐GSDMD pathway can mitigate MS severity, surpassing first‐line MS drugs and underscoring CASP1 potential as a therapeutic target.^48^ Its consistent upregulation and functional role suggest it may play a central role in sustaining MS WM inflammation.

Our approach is limited, primarily by the lack of access to raw data for many studies, which restricts our ability to assign precise confidence measures to individual genes. We could rank genes by sample size and average fold change, but we could not provide a true combined fold change or *p*‐value. This highlights the necessity for researchers to make raw data available to apply more robust meta‐analysis methods. Notably, the lack of significance for the AL versus control WM gene overlap, which relied mainly on published lists with minimal raw data, illustrated the significant impact of data availability on identifying consistent gene expressions across studies. Improved data sharing and standardized experimental methods could substantially enhance the robustness and reproducibility of future research.

Further, while our study aimed to improve specificity by focusing on consistently deregulated genes across multiple studies, we acknowledge that this approach does not necessarily capture all biologically relevant factors. A limitation intrinsic to gene expression studies is that mRNA levels may not directly correlate with protein abundance or activity, especially where post‐translational modifications, protein stability, or enzyme activation are critical in MS pathology. Consequently, some genes integral to MS processes might remain undetected, potentially as “false negatives,” highlighting the importance of incorporating proteomic or post‐translational data for a more comprehensive understanding of disease mechanisms.

In conclusion, despite the aforementioned limitations, our research establishes a solid methodology for integrating gene expression data, effectively identifying cellular and molecular mechanisms underlying human diseased tissue, with significant potential for translating these findings into drug development strategies.

## Author Contributions

G.A., A.I., M.R., and R.N. contributed to the conception and design of the study. G.A., A.I., O.H., A.P., and P.S. contributed to the acquisition and analysis of data. G.A., A.P., S.B., R.M., S.H., R.R., M.R., and R.R contributed to drafting the text or preparing the figures.

## Potential Conflicts of Interest

No conflict of interest reported by the Authors in relation to this manuscript.

## Supporting information


**Table S1.** Syntax used for database search on Scopus and PubMed July 2024.

**Table S6.** Gene ranking multiple sclerosis (MS) white matter (WM) versus control WM.
**Table S7.** Gene ranking normal appearing white matter (NAWM) versus control white matter (WM).
**Table S8.** Gene ranking active lesions (AL) versus control white matter (WM).
**Table S9.** Gene ranking chronic active lesions (CAL) versus control white matter (WM).

**Table S11.** Gene set enrichment analysis‐multiple sclerosis (MS) white matter (WM) versus control (upregulated genes).
**Table S12.** Gene set enrichment analysis‐multiple sclerosis (MS) white matter (WM) versus control (downregulated genes).
**Table S13.** Gene set enrichment analysis‐normal appearing white matter (NAWM) versus control (upregulated genes).
**Table S14.** Gene set enrichment analysis‐active lesions (AL) versus control (upregulated genes).
**Table S15.** Gene set enrichment analysis‐chronic active lesions (CAL) versus control (upregulated genes).
**Table S16.** Gene set enrichment analysis‐chronic active lesions (CAL) versus control (downregulated genes).

**Table S17.** Multiple sclerosis (MS) white matter (WM) versus control (Cluster 1).
**Table S18.** Multiple sclerosis (MS) white matter (WM) versus control (Cluster 2).
**Table S19.** Multiple sclerosis (MS) white matter (WM) versus control (Cluster 3).
**Table S20.** Multiple sclerosis (MS) white matter (WM) versus control (Cluster 4).
**Table S21.** Multiple sclerosis (MS) white matter (WM) versus control (Cluster 5).
**Table S22.** Multiple sclerosis (MS) white matter (WM) versus Control (Cluster 6).
**Table S23.** Active lesions (AL) versus control (Cluster 1).
**Table S24.** Chronic active lesions (CAL) versus control (Cluster 1).
**Table S25.** Chronic active lesions (CAL) versus control (Cluster 2).

**Table S26.** Multiple sclerosis (MS) white matter (WM) versus control white matter (WM) (Cluster 1).
**Table S27.** Multiple sclerosis (MS) white matter (WM) versus control white matter (WM) (Cluster 2).
**Table S28.** Multiple sclerosis (MS) white matter (WM) versus control white matter (WM) (Cluster 3).
**Table S29.** Multiple sclerosis (MS) white matter (WM) versus control white matter (WM) (Cluster 4).
**Table S30.** Multiple sclerosis (MS) white matter (WM) versus control white matter (WM) (Cluster 5).
**Table S31.** Multiple sclerosis (MS) white matter (WM) versus control white matter (WM) (Cluster 6).
**Table S32.** Active lesions (AL) versus control white matter (WM) (Cluster 1).
**Table S33.** Chronic active lesions (CAL) versus control white matter (WM) (Cluster 1).
**Table S34.** Chronic active lesions (CAL) versus control white matter (WM) (Cluster 2).

**Table S35.** Published in vivo model validation.

**Table S1.** Syntax used for database search on Scopus and PubMed July 2024.

## Data Availability

The gene lists for each comparison for each study, extracted, mapped, and filtered based on fold change (raw data), as well as the code used for this study are deposited at https://github.com/gianmarcoabbadessa/Gene-expression-meta-analysis-codes.
