# Supplementary figures and images for: Mapping Molecular Pathways of Multiple Sclerosis: A Gene Prioritization and Network Analysis of White Matter Pathology Transcriptomics
Source: Ann Neurol. 2025 Feb 14;98(1):67–79. doi: 10.1002/ana.27216 (PMC12174739; doi:10.1002/ana.27216)

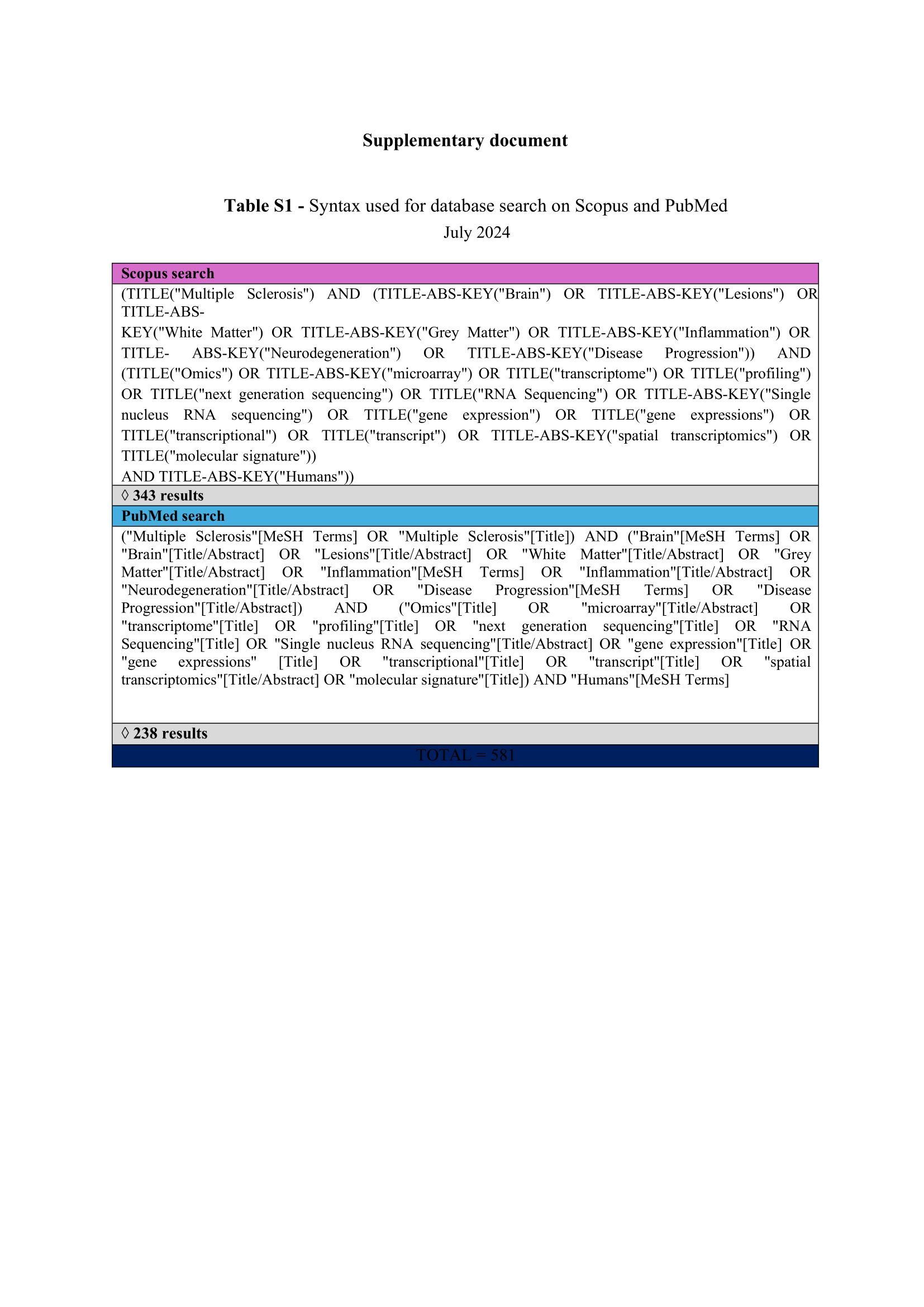

Supplement: Supplementary file 1 — Table S1. Syntax used for database search on Scopus and PubMed July 2024. [file ANA-98-67-s006.tiff]
