# Supplementary material for: Mapping Molecular Pathways of Multiple Sclerosis: A Gene Prioritization and Network Analysis of White Matter Pathology Transcriptomics
Source: Ann Neurol. 2025 Feb 14;98(1):67–79. doi: 10.1002/ana.27216 (PMC12174739; doi:10.1002/ana.27216)
Supplement: Supplementary file 7 — Table S1. Syntax used for database search on Scopus and PubMed July 2024. [file ANA-98-67-s003.docx]

**Supplementary Methods**

*Gene universe definition*

The gene universe corresponds to the list of all gene tested across the nine included studies. Given the unavailability of comprehensive gene testing data for some studies, we adopted the following procedure: four studies ^10-12, 16^ had deposited raw data in GEO; and for these studies, we retrieved the list of gene symbols from the expression matrix present in GEO. For studies using Affymetrix platforms ^6, 9^, we extracted the list of gene symbols related to that platform from other studies using same or similar platforms (Affymetrix Human Genome U95A Array and Affymetrix Human Genome U133A Array). Finally, for studies with custom arrays, the appropriate number of genes was chosen from the combined gene list of all other platforms (mirroring the approach of Griffith et al. 2006^17^). More details regarding GEO access numbers, platform type and size are reported in Table S5. The resulting gene universe consisted of 45,478 genes. However, this list did not include expression values for the WM samples and could only have been utilized for Monte Carlo simulations with random sampling.

To account for gene co-expression dependencies in the simulations we needed a gene universe comprehensive of gene expression values relative to the included samples. Indeed, we recognized that gene dependencies to be considered should reflect those observed in the datasets included in this integrative analysis (and thus MS-related). These data reflect the local biology of the MS condition and tissue-specific transcriptional phenomena, making them highly relevant for the analysis. However, calculating gene co-expression across all studies, to comprehensively reflect gene-gene dependencies, would be ideal but ultimately unfeasible. This limitation arises because raw gene expression data were available for only a few studies, and integrating data from studies using varying sequencing strategies would compromise consistency and reliability. Given this, we based our co-expression analysis on the data from the study by Elkjaer et al. (2019; GSE138614)^16^, the largest dataset among those included (78 samples from 15 donors). This study used RNA sequencing on a broad range of tissue types (normal-appearing white matter [NAWM], active lesions [AL], chronic active lesions [CAL], and control white matter), providing a robust basis for our network construction. Additionally, the Elkjaer dataset provided a broad genomic landscape, closely matching the gene universe defined above. It includes 39,378 genes mapped to unique Entrez IDs, representing 85.6% of the 45,478 transcripts tested across the nine studies, and captures 5,174 of the 5,201 (99.46%) DEGs identified across the studies, including all 528 multi-study DEGs (100%). Therefore, it is highly suitable as surrogate of the gene universe in the simulations, for analyses requiring adjustment of gene co-expression dependencies.

*Weighted Gene Co-expression Network Analysis*

The TPM dataset (GSE138614) originally consisted of 98 samples. Twenty samples corresponding to inactive and remyelinated lesions, which were not included in this study, were removed. The remaining 78 samples were derived from 15 subjects. Each subject contributed a varying number of samples.

Genes with zero variance across samples were excluded, reducing the total number of genes from 39,376 to 38,705, Therefore, genes with meaningful variability were retained while maintaining a representative gene universe. After log2 transforming the expression matrix to stabilize variance and minimize the influence of low expression values, we scaled and centred, standardizing expression levels across genes to enable the comparability of co-expression relationships.

To account for intra-subject variability and prevent biases due to unequal donor representation, we applied a Linear Mixed Model (LMM) to the expression matrix for each gene using the formula Expression ~ 1 + (1 | Individual_ID). The residuals, representing expression values adjusted for donor-specific effects, were extracted and used for downstream analyses. This correction effectively mitigated the influence of sample imbalance per donor and ensured the results reflected biological differences rather than donor-specific variability.

We used the WGCNA R package^23-25^ to explore the gene co-expression network. For the construction of a signed scale-free network, we applied the WGCNA *adjacency()* function with a soft-thresholding power of 12 (default value for achieving scale-free topology in signed network type). The signed network type was selected to focus on positive correlations, as these are more likely to capture biologically meaningful co-expression relationships that could explain the observed overlap of DEGs across studies. We next defined a topological overlap matrix using the *TOMsimilarity()* function, which accounts for both **direct connections** and **shared neighbors**, capturing higher-order relationships within the network and identifying genes with similar expression patterns*.* To quantify the relative connection of each gene in the network, we calculated TOM-based whole-network connectivity scores by summing the topological overlap scores for each gene across all others in the network.

We chose TOM-based connectivity on the whole network as weights for the simulations because it provides a robust measure of gene interconnectedness by incorporating both direct and indirect interactions. This approach captures the topological overlap across the entire network, ensuring a more comprehensive representation of gene connectivity compared to intramodular metrics, which are module-specific and less suitable for global comparisons with observed overlaps across studies.

These scores were normalized to generate a vector of gene weights, representing the relative TOM-based whole-network connectivity of each gene within the scale-free network. These weights were then incorporated into Monte Carlo simulations, where they influenced the probability of sampling each gene. By incorporating the WGCNA, resampling in the Monte Carlo simulation was not performed with equal probability across all genes. Instead, it was weighted according to co-expression dependencies derived from gene connectivity in the network. This weighting accounted for the likelihood of a gene being part of the observed biological structure, ensuring that the simulations respected the natural dependencies inherent in the network.

**Sensitivity analysis**

To minimize the influence of studies with small sample sizes, RNA extracted from formalin-fixed tissues, insufficiently detailed statistical analyses, or other factors considered in the BIOCROSS quality assessment, we performed the vote-counting strategy and Monte Carlo simulation, excluding the four studies that scored less than 20/29 in the BIOCROSS quality control ^4, 6, 10, 14^ (Supplementary file, Table S2). These excluded studies contributed a minimal number of significant genes; indeed, the number of overlapping genes decreased from 528 to 492, yet the results remained highly significant in the Monte Carlo simulation (p < 0.0001).
